# Water nanostructure formation on oxide probed in situ by optical resonances

**DOI:** 10.1126/sciadv.aax6973

**Published:** 2019-10-25

**Authors:** Yin Yin, Jiawei Wang, Xiaoxia Wang, Shilong Li, Matthew R. Jorgensen, Junfeng Ren, Sheng Meng, Libo Ma, Oliver G. Schmidt

**Affiliations:** 1 Institute for Integrative Nanosciences, Leibniz IFW Dresden, Helmholtzstr. 20, 01069 Dresden, Germany.; 2 Material Systems for Nanoelectronics, Chemnitz University of Technology, Reichenhainer Str. 70, 09107 Chemnitz, Germany.; 3 School of Physics and Electronics, Shandong Normal University, 250014 Jinan, China.; 4 Beijing National Laboratory for Condensed Matter Physics and Institute of Physics, Chinese Academy of Sciences, Beijing 100190, China.; 5 Research Center for Materials, Architectures and Integration of Nanomembranes (MAIN), Rosenbergstraße 6, TU Chemnitz, 09126 Chemnitz, Germany.; 6 Nanophysics, Faculty of Physics, TU Dresden, 01062 Dresden, Germany.

## Abstract

The dynamic characterization of water multilayers on oxide surfaces is hard to achieve by currently available techniques. Despite this, there is an increasing interest in the evolution of water nanostructures on oxides to fully understand the complex dynamics of ice nucleation and growth in natural and artificial environments. Here, we report the in situ detection of the dynamic evolution of nanoscale water layers on an amorphous oxide surface probed by optical resonances. In the water nanolayer growth process, we find an initial nanocluster morphology that turns into a planar layer beyond a critical thickness. In the reverse process, the planar water film converts to nanoclusters, accompanied by a transition from a planar amorphous layer to crystalline nanoclusters. Our results are explained by a simple thermodynamic model as well as kinetic considerations. Our work represents an approach to reveal the nanostructure and dynamics at the water-oxide interface using resonant light probing.

## Teaser

Dynamics of water nanostructure formation on oxide surface is probed in situ by optical resonances.

## INTRODUCTION

As a ubiquitous phenomenon, the nanostructure and dynamics of water adsorbates on solids have attracted huge interest in many research fields including electrochemistry, corrosion, heterogeneous catalysis, and environmental sciences. Over the past decades, numerous studies have been dedicated to examining the behavior of water nanolayers on solid surfaces under vacuum conditions and at low temperature (*1*–*5*). However, experimental measurements were performed exclusively on single-crystal metal substrates because of a very limited number of available surface detection techniques, such as scanning tunneling microscopy (STM), which requires conducting substrates (*6*–*8*), and low-energy electron diffraction (LEED), which requires crystal periodicity (*3*, *9*). In contrast to single-crystal metals, amorphous oxides are abundant in nature and are of fundamental importance in materials science and applications. However, investigation of the nanostructure and dynamics of water adsorbates on pure oxide surface has been elusive by using conventional techniques (e.g., STM and LEED) due to the materials’ high–dielectric constant and/or amorphous structure. Although sum frequency spectroscopy has been developed to detect local chemical bond vibrations at oxide interfaces (*10*, *11*), this technique has major difficulties in detecting mesoscopic structural changes in molecular layers, and measuring the nanostructure variations of water monolayers is even more difficult.

Previous reports have often focused on only the first few adsorbed layers forming two-dimensional (2D) islands (*2*, *5*, *12*). There is, nevertheless, an increasing interest in investigating water multilayers to understand the full formation dynamics, especially the conversion between planar layers and 3D nanoclusters of this complex system on an oxide surface. Thus, exploring new techniques suitable for the in situ detection of the nanostructure and dynamics of water multilayers on oxides, in particular amorphous dielectric oxides, is urgently needed and of high relevance for basic science and potential applications.

Here, the nanostructure and dynamics of water multilayers on an amorphous HfO_2_ surface are investigated by optical whispering gallery mode (WGM) resonances in a microtube cavity. Optical WGM microcavities are known to be extremely sensitive to surface perturbations, allowing the detection of objects down to single organic molecules or nanoparticles (*13*–*15*). We prepare microtube optical cavities by the roll-up of prestrained nanomembranes on a silicon substrate (*16*). Resonant light is guided around the microtube to form WGM resonances in the visible spectral range (*17*), and the evanescent field is used for surface sensing (*14*). The spectral position and width of the resonant mode are monitored to reveal the nanostructure evolution of the water multilayer throughout the growth-desorption processes on the oxide surface of the cavity, which is controlled by tuning the system temperature. Perturbation theory is applied to quantitatively analyze the surface water adsorption on WGM microcavities (*18*–*20*). Our investigation shows that a water multilayer grows in the form of nanoclusters initially and continues as a planar film in the following. During desorption, a conversion of the planar layer into a cluster morphology is observed because of thermal activation. The observed phenomena are explained by minimization of surface energy carried out in the framework of a thermodynamic equilibrium model and are further verified by kinetic considerations.

## RESULTS

**Fig. 1:**
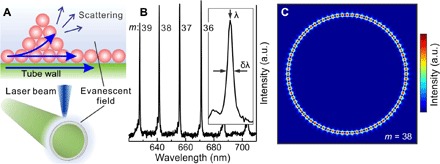
The evanescent field of resonant light in a microtube cavity is sensitive to surface molecular layers and/or nanoclusters. (**A**) The bottom panel shows schematically a microtube cavity being excited by a laser beam. The top panel shows a schematic of resonant light propagating in the tube wall and the surface molecular layer, being scattered by molecular clusters. (**B**) Measured optical resonant mode spectrum labeled by mode numbers *m* = 36 to 39. The inset shows an individual mode where the mode shift characterizes the thickness of the molecular layer, and the variation of the mode width (δλ) characterizes the light scattering by the surface clusters. (**C**) Electric field profile of mode *m* = 38 taken to quantify the resonant mode shift induced by adsorbed molecular layers. a.u., arbitrary units.

The surface sensing ability relies on the interaction between the surface molecules and the evanescent field of resonant light at the tube surface. This interaction leads to two remarkable effects: (i) The resonant evanescent light field is modified because of the increase/decrease of the molecule layer thickness, and (ii) the resonant light is scattered by nanoclusters on the tube surface, as schematically shown in the top panel of Fig. 1A. The bottom panel of Fig. 1A shows a sketch of a microtube cavity being excited by a laser, and Fig. 1B shows the optical resonant mode spectrum (see Materials and Methods). Effect (i) results in a spectral shift of the resonant modes (*14*), while effect (ii) leads to a broadening of the resonant modes (*21*). Hence, this technique collects valuable information about the nanostructure evolution of surface objects without the requirement of direct imaging. Because all the resonant modes respond in the same way to the presence of surface objects, the evolution of only one resonant mode (e.g., mode number *m* = 38) is discussed in the following. Figure 1C shows the calculated electric field for resonant mode *m* = 38. On the basis of this simulation, a perturbation theory analysis is carried out to calculate the mode shift induced by the presence of a molecular layer on the tube surface (see the Supplementary Materials) (*18*–*20*). In this analysis, the presence of the thin molecular layer leads to a mode shift

$$\Delta \omega =-\frac{\omega }{2}\frac{\langle E(\vec{r})|\Delta \varepsilon (\vec{r})|E(\vec{r})\rangle }{\langle E(\vec{r})|\varepsilon (\vec{r})|E(\vec{r})\rangle }$$ (1)

where ω, $E(\vec{r})$, and $\varepsilon (\vec{r})$ are the resonance angular frequency, the electric field distribution in the resonator, and the permittivity, respectively. $\Delta \varepsilon (\vec{r})$ denotes the permittivity variation induced by the surface molecular layers.

**Fig. 2:**
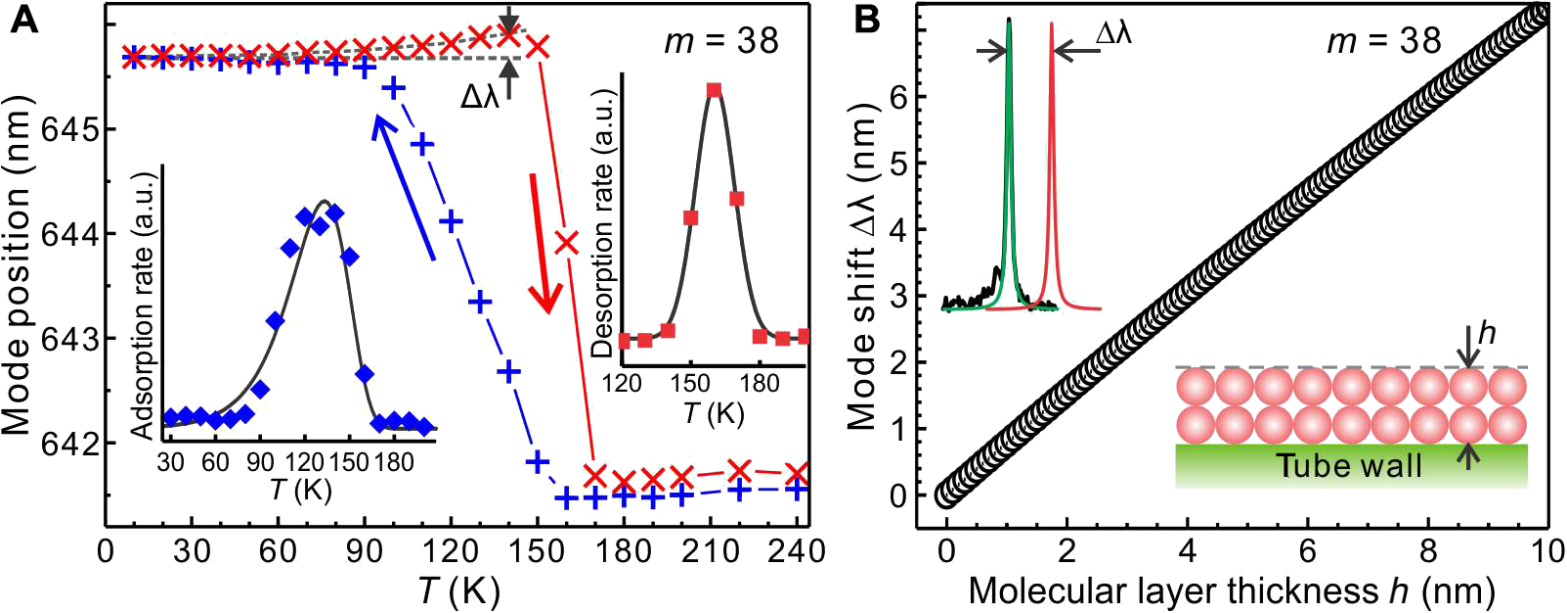
Evolution of resonant mode position throughout the water layer growth and desorption processes. (**A**) Optical mode (*m* = 38) shift during water layer growth (blue plus symbols) and desorption (red cross symbols) processes triggered by decreasing and increasing the temperature, respectively. The adsorption/desorption rate deduced from the differential derivative of the adsorption/desorption curve is shown in the left-bottom/right-top inset. (**B**) Mode (*m* = 38) shift induced by surface water layer (*h*) calculated by perturbation theory. The insets show the schematic of the resonant mode shift (top left) due to the presence of a molecular layer on the tube surface (bottom right).

Figure 2A shows the evolution of the resonant mode as a function of temperature, representing the growth and desorption processes of a water multilayer on the microtube surface (see Materials and Methods). The mode position remains constant, while the temperature decreases from room temperature to 160 K. This indicates that water layer growth is absent on the tube surface, which is explained by a desorption rate that is larger than the adsorption rate at temperatures >160 K as previously reported under similar vapor pressure (*22*). When the temperature falls from 160 to 90 K, the optical mode redshifts continuously, indicating successive water layer growth on the tube surface. This is caused by the increased/decreased adsorption/desorption rate during the descending temperature. During this process, the pressure drops from 10^−6^ to 10^−8^ mbar. The adsorption rate is obtained by a differential analysis of the mode shift [equivalent to a thickness change of the molecular layer (*14*)] curve (see inset of Fig. 2A). From 90 to 10 K, the mode spectral position remains constant, implying that water layer growth has ceased, i.e., the residual water molecules in the vacuum chamber do not lead to any noticeable water layer growth. On the basis of perturbation calculations, the mode shift is proportional to the molecular layer thickness as plotted in Fig. 2B. The measured total mode shift is 4.19 nm, which corresponds to adhesion of a ~5.4-nm-thick water layer [~18 monolayers (MLs)].

To study water molecule layer desorption, the system temperature is increased from 10 K to room temperature. The mode position remains stable from 10 to 50 K, confirming the stability of the water nanolayers on the tube surface. The optical mode does not start blueshifting for *T* > 90 K, which would be the reverse of the adsorption process. Instead, the optical mode redshifts by Δλ ~ 0.18 nm (corresponding to ~0.23-nm change in water layer thickness) when the temperature increases from 50 to 140 K, indicating a volume expansion of the water multilayer. The observed volume expansion is around 10 times larger than that of bulk ice Ih (*23*). This implies that a phase transition, rather than a simple thermal expansion of the film, is involved in the process, which will be discussed later in the text. A substantial blueshift is observed when the temperature is raised beyond 140 K, where the water multilayer is destabilized by hydrogen bond breaking (*3*). The peak of the water layer desorption rate located at ~160 K is deduced from a differential analysis of the desorption curve (see inset of Fig. 2A). The measured layer desorption temperature (~160 K) is in agreement with that on metals using temperature programmed desorption (TPD) (*3*, *24*, *25*), indicating a weak dependence of the substrate for water multilayer desorption. The optical mode rapidly shifts back to the initial spectral position for *T* > 170 K, documenting the entire desorption of the water nanolayers while the pressure returns back from 10^−8^ to 10^−6^ mbar.

The processes observed above are described by a hysteretic behavior, implying different dynamics for water layer growth and desorption. In this context, it should be noted that our approach is different from the conventionally used TPD technique for detecting molecular layer changes on solid surfaces (*3*, *24*, *25*). The TPD technique measures the ionized species flying into a mass spectrometer chamber, which provides only little information about the molecular layer dynamics on the sample surface.

**Fig. 3:**
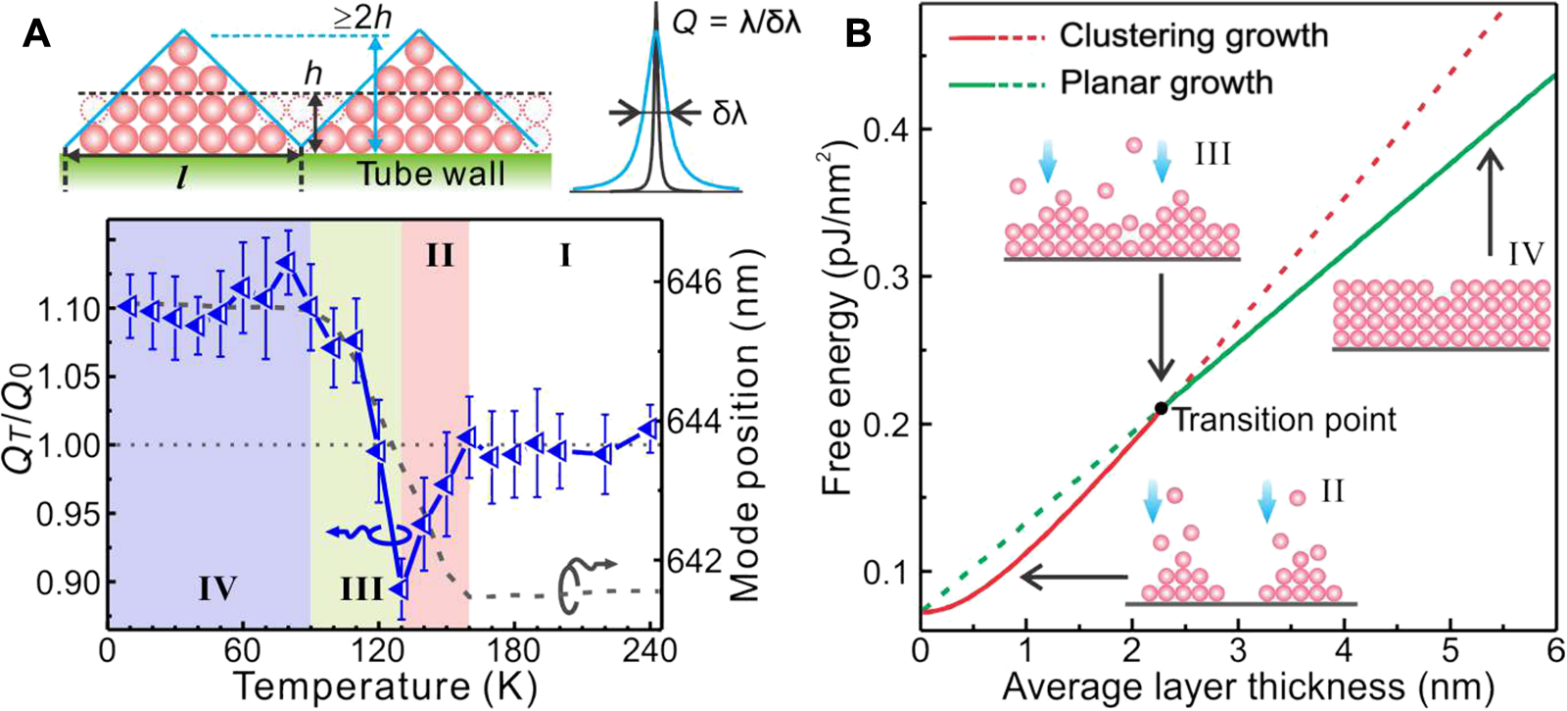
Water layer growth dynamics probed during decreasing system temperature. (**A**) Four regions (I to IV) are identified by the comparison between *Q* factor variation (blue triangle symbols) and mode shift (black dashed line). The error bars represent the SEs in fitting the mode measured at different temperatures. The inset (top) shows a schematic of nanoclusters and planar layer formed by the same amount of adsorbed molecules. While the two morphologies result in the same spectral mode shift, the nanoclusters lead to a broadened peak (decreased *Q* factor), and the smoothing planar layer results in a narrowed peak (increased *Q* factor). (**B**) Free energy in nanocluster (red curve) and planar (green curve) morphology as a function of average layer thickness. The adsorption selects the growth mode with the lowest energy in each region. Schematic of the water layer structure in region II (clustering growth), region III (turning to planar growth after reaching the transition point), and region IV (formation of a planar layer).

To investigate the nanostructure evolution of the water multilayer, we analyze the variation of the *Q* factor, i.e., the inverse mode width relative to its mode frequency (*Q* = λ*/*Δλ). Figure 3A shows the evolution of *Q_T_*/*Q*_0_ as a function of temperature, where *Q*_0_ is the initial *Q* factor and *Q_T_* denotes the *Q* factor measured at temperature *T*. The mode spectral position measured during the water layer growth process (as shown in Fig. 2A) is also plotted in the same graph for direct comparison. Four regions (I to IV) can be identified by correlating the *Q* factor variation with the optical mode shift, where the *Q* factor reflects certain changes in layer morphology and the optical mode shift denotes the changes in average layer thickness.

Region I spans from room temperature to 160 K, where both the *Q* factor and mode position remain stable. A notable *Q* factor decrease is observed in region II (160 to 130 K), where the mode position starts to redshift. This suggests that, instead of planar growth, the formation of nanoclusters occurs on the oxide surface of the tube cavity. It is expected that water is less likely to cluster on oxides because the polar nature of an oxide surface facilitates stronger water binding than on metal surfaces where 2D and/or 3D water clustering has been observed (*6*, *26*). However, our results show that at the beginning of the adsorption process, the formation of nanoclusters is favored on a polar amorphous HfO_2_ surface. In a kinetic view, it has been reported that adsorbed water molecules are relatively mobile on a surface, allowing for the formation of crystalline nanoparticles at high adsorption temperatures (≥135 K) (*3*, *24*), similar to the temperature of region II. Our results therefore imply that the adsorbed molecules diffuse and aggregate into ice clusters on the HfO_2_ surface at adsorption temperatures >130 K.

In region II, the mode shifts by 1.83 nm, corresponding to an average thickness of 2.3 nm (~7.8 MLs) of the adsorbed layer. Converting this value into cluster morphology, the average height of the clusters is estimated to be ≥15 MLs (see Fig. 3A), assuming a shape of the ice clusters previously reported in (*2*). In region III (130 to 90 K), the optical mode continues to redshift, indicating the continuation of the water layer growth. In this region, the *Q* factor increases substantially, indicating that progressively adsorbed water molecules tend to adhere to the periphery of the nanoclusters, thereby filling up the gaps between the clusters and recovering planar growth. In previous reports, a layer-by-layer growth behavior has been reported at low adsorption temperature (<120 K) to grow an amorphous water nanofilm on Pt(111) (*3*, *27*), in which the temperature matches well with our region III. Eventually, the *Q* factor turns out to be higher than its initial *Q*_0_ and reaches *Q_T_*/*Q*_0_ ~1.11 in region IV (90 to 10 K), which demonstrates that the water nanofilm becomes smoother than the original amorphous HfO_2_ surface. The roughness of the initial tube surface is around 1 nm (see the Supplementary Materials); thus, the water nanofilm assumes an atomically smooth surface at the end. Hence, a clear changeover from clustering to planar growth is revealed when decreasing the temperature.

The transition from clustering to planar growth can be explained by a simplified thermodynamic model. As labeled in Fig. 3A, the average film thickness is *h*, and the average surface morphology oscillation length is *l* in two dimensions on the HfO_2_ surface. For the flat film, the free energy in an *l* × *l* square unit is the sum of surface energy and film strain energy

$$E_{\text{flat}}=l^{2}\gamma +{\mathrm{hl}}^{2}\sigma$$ (2)

where γ denotes surface tension of the film and σ denotes stress in the planar film. For simplicity, the ice clusters are assumed to take a pyramidal shape, which has been reported elsewhere (*28*). Since clusters always have a larger surface area compared with that of corresponding planar film, our model and conclusion can also be applied to other cluster forms (*29*). In addition, only surface energy is considered for the clusters because the strain energy is mostly released in isolated clusters/islands (*28*). To let a pyramid and a planar film have the same volume within the same surface unit *l* × *l*, i.e., $\frac{l^{2}t}{3}={\mathrm{hl}}^{2}$, the following holds: *t* = 3*h*, where *t* is the height of the pyramid. The energy for a pyramid therefore reads

$$E_{\text{rough}}=4\gamma \times \frac{l}{2}\times \sqrt{{\left(\frac{l}{2}\right)}^{2}+{\left(3h\right)}^{2}}$$ (3)

From this, we can easily derive that when the effective film thickness *h* is larger than a certain critical thickness $h_{c}\equiv \frac{x}{9{\left(\frac{x}{l}\right)}^{2}-1}$, where$\;x=\frac{2\gamma }{\sigma }$, the energy for the flat film is smaller than that for the cluster (*E*_flat_ ≤ *E*_rough_). In other words, when the film thickness reaches a critical value *h*_c_, instead of clustering, a flat layer growth is preferred to reduce surface energy. In our measurement, the clustering ceased and turned to planar growth when the average thickness reaches 2.3 nm. This value can be well fitted by adopting the following parameters: γ = 0.072 N/m, an ice bulk modulus of 11 GPa, a small strain of 0.3045% (i.e., σ = 0.0609 GPa), and *l* = 5 nm, which are in agreement with values reported previously for ice (*23*). With these parameters, Eqs. 1 and 2 are plotted in Fig. 3B as a function of grown average water layer thickness, where the form of the water layer in each region and the morphology transition are identified. We note that the interface energies between the oxide and water contact area are not taken into account because they mostly cancel each other for continuous and quasi-continuous water layers, and the bulk property of stress energy in flat water films dominates in Eq. 2.

**Fig. 4:**
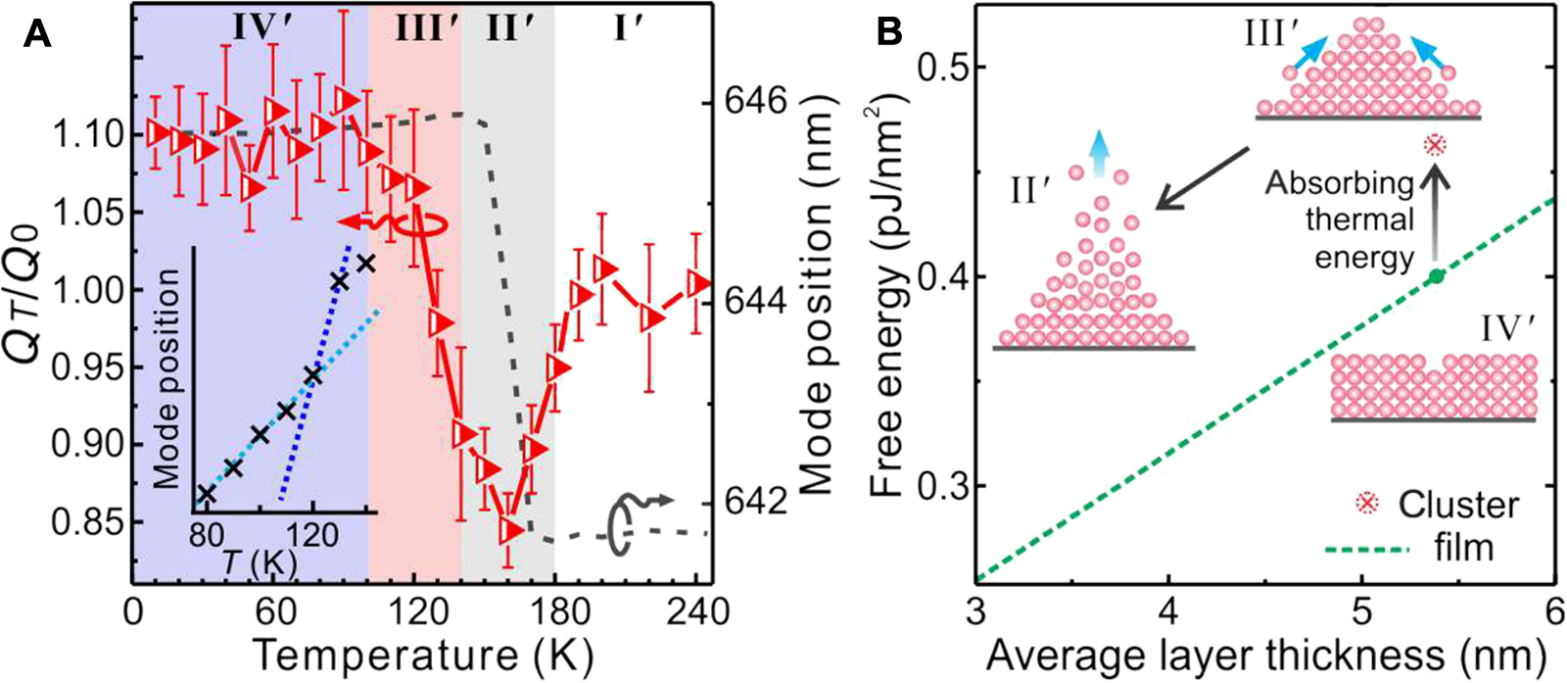
Water layer desorption dynamics probed during increasing system temperature. (**A**) Four regions (I′ to IV′) are identified by the comparison between *Q* factor variation (red triangle symbols) and mode shift (black dashed line). The error bars represent the SEs in fitting the mode measured at different temperatures. The inset shows a nonlinear mode shift in the range of 80 to 140 K. (**B**) Upon heating up, the water film rearranges from the planar morphology in region IV′ to the cluster morphology in region III′ and eventually desorbs in region II′.

Four regions are also identified in the temperature-activated desorption process, as shown in Fig. 4A. In region IV′ (10 to 100 K), the *Q* factor stays constant, suggesting that the water multilayer remains stable. In region III′ (100 to 140 K), the *Q* factor starts to decrease while the mode position remains almost constant. This indicates that in this region, the molecules do not desorb from but instead become mobile on the surface and start nucleating into nanoclusters. The top water layer adsorbed at low temperature (ca. <100 K) stays amorphous because of insufficient molecule mobility (*3*). As temperature increases (>125 K), water molecules become more mobile and aggregate into crystalline nanoclusters (*3*). We interpret this result as a phase transition from a dense amorphous phase to a low-density crystal phase, which explains the abnormal volume increase observed by the spectral redshift in Fig. 2A. The phase transition is further supported by the nonlinear mode shift during the temperature increase, as shown in Fig. 4A. This phenomenon can also be explained by our thermodynamic model (see Fig. 4B). In the heating-up process, the flat film becomes unstable because of thermal activation. The film starts to undergo cluster coarsening, which accommodates the heat thermal energy. In region II′ (140 to 180 K), the water nanolayer begins to desorb because the molecular vibration energy is sufficient to overcome the hydrogen bonding in the water nanoclusters. The pronounced desorption of the water nanoclusters generates an even rougher surface, which results in a minimum *Q* factor in this region. In region I′, both the *Q* factor and the mode position return to their initial values.

## DISCUSSION

**Fig. 5:**
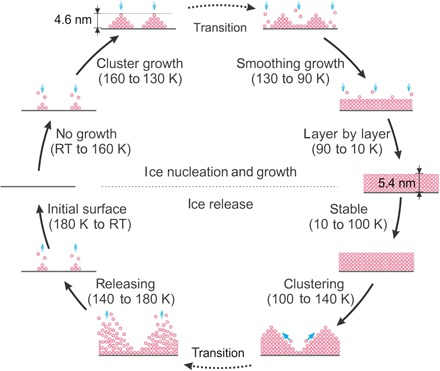
Overview of water layer dynamics in the growth and desorption processes. The dynamics of water layer growth/desorption processes are detected in situ over different temperature regimes. RT, room temperature.

Figure 5 shows an overall view of the growth and desorption processes of the water layer structures on an oxide (HfO_2_) surface accessible by varying the temperature in a vacuum chamber. Our measurement results show that the water molecules start to adsorb on the oxide surface at a temperature of 160 K, where clustering growth occurs in the temperature range of 160 to 130 K. In the range of 130 to 90 K, the water layer turns to planar growth as the adsorbing water molecules become less mobile on the surface and, consequently, tend to adhere to the periphery of the water nanoclusters to get a minimum of strain energy. Since water molecules are still adsorbing from 130 to 90 K, the planar film growth might be governed by kinetics rather than thermodynamics. In the low-temperature range of 90 to 10 K, layer-by-layer growth is observed. In contrast to the growth process, in the desorption process, the flat layer remains stable when the temperature is lower than 100 K. In the temperature range between 100 and 140 K, the water molecules diffuse and aggregate into crystalline water clusters. The film roughens spontaneously well before any water desorbs, clearly indicating that it is thermodynamically driven. When the temperature is increased from 140 to 180 K, the water molecules get released by hydrogen bond breaking. In the temperature range from 180 K to room temperature, the water molecules are fully desorbed and the substrate recovers to its initial condition. As a result, with our approach, the successive evolution of water layer formation and dynamics in the growth and desorption processes can be in situ probed in real time, which has been illusive by previously reported techniques.

Our work provides a versatile platform to investigate surface molecular dynamics at both low and room temperature. In addition, by modifying the tube surface, it is also possible to study molecular dynamics on both hydrophobic and hydrophilic surfaces, as well as on metallic surfaces. Our method also allows for measuring phase transition in molecular layer by further improving the *Q* factor (i.e., a sharper mode) and experimental design (i.e., no notable molecule adsorption/desorption during the phase transition). Currently, it is difficult to identify the exact facets of ice crystals from the present measurement. Revealing this information might be possible if the new technique is further developed to include the anistropic dielectric constant of crystals into consideration and/or combined with other techniques such as x-ray diffraction. Last, we note that although the cryostat vacuum chamber was flushed with water vapor, some other minor species, such as H_2_ and CO, might be involved in the adsorption. However, H_2_ adsorption on oxide occurs at low temperature <35 K in raw vacuum (*30*), while that of CO starts at 300 to 200 K (*31*), where we did not observe any notable change of the resonant mode. Thus, we rate these concentrations smaller than what would be required to substantially influence the adhesion between water and the substrate (*1*).

In conclusion, this work investigates the nanostructure and dynamics of water multilayers on amorphous oxide surfaces in situ that occur during the growth and desorption processes. In contrast to previous studies where water nanostructures with submonolayer to bilayer coverage were investigated, here the nanostructure evolution of a water film up to ~18 MLs is observed by optical means. Our report addresses fundamental questions regarding the nanostructure and dynamics of molecular films on oxide, for example, to reveal how a water multilayer grows and desorbs as it is thermally activated. Our work paves the way for understanding the nanostructure and dynamics of water thin films on oxide surfaces relevant for both fundamental and applied research.

## MATERIALS AND METHODS

### Fabrication of optical microtube cavities

Optical microtube cavities (~5 μm in diameter) were prepared by self-rolling of prestrained SiO*_x_* nanomembranes (~40 nm in thickness). As previously described in detail (*17*), electron beam evaporation was used to deposit SiO*_x_* nanomembranes on predesigned circular sacrificial patterns. By dissolving the sacrificial pattern, the SiO*_x_* rolls up into a microtubular structure to release the nanomembrane strain. Afterward, atomic layer deposition (*32*) was used to coat the tube surface with a 30-nm-thick HfO_2_ layer, which has a high dielectric constant and experiences strong polar bonding (*33*).

### Experimental setup for in situ measurement

Following a commonly used strategy (*1*, *2*), the microtube was placed into a vacuum chamber (~10^−6^ mbar) that contained water molecules as the main residual gas even without water vapor flushing. In our experiment, the water vapor flushing can further increase the concentration of H_2_O molecules. The temperature was regulated in a liquid helium cryostat to tune water adsorption and desorption on the tube surface. A 532-nm laser line was used to excite the nonbridging oxygen hole center defects in the tube wall, which emit light in the visible spectral range (*34*). In this setup, a 50× objective lens was used to focus the excitation laser beam onto the tube wall and collect the resonant spectral signal emitted from the microtube. The optical WGM modes, originating from the self-interference of light circulating in the thin tube wall, were recorded through a laser confocal detection setup. The temperature was changed with a rate of around 2 K/min to go from one measurement temperature point to the next. At each measurement temperature point, the resonant mode was recorded after the system reached a temporary quasi-equilibrium (taking around 10 min) between molecular adsorption and desorption.

## Supplementary Material

http://advances.sciencemag.org/cgi/content/full/5/10/eaax6973/DC1

Download PDF

Water nanostructure formation on oxide probed in situ by optical resonances

## SUPPLEMENTARY MATERIALS

Supplementary material for this article is available at http://advances.sciencemag.org/cgi/content/full/5/10/eaax6973/DC1 Section S1. Perturbation theory analysis Section S2. Quality factor variation versus surface roughness Section S3. Surface morphology of HfO_2_ Fig. S1. Measured and simulated WGM resonances in a microtubular cavity. Fig. S2. Quality factor variation (*Q_T_*/*Q*_0_) as a function of surface roughness. Fig. S3. Surface morphology of HfO_2_ characterized by SEM and AFM. Reference (*35*)
