# Supplementary material for: Water nanostructure formation on oxide probed in situ by optical resonances
Source: Sci Adv. 2019 Oct 25;5(10):eaax6973. doi: 10.1126/sciadv.aax6973 (PMC6814375; doi:10.1126/sciadv.aax6973)
Supplement: http://advances.sciencemag.org/cgi/content/full/5/10/eaax6973/DC1 [file supp_5_10_eaax6973__index.html]

Science Advances | Science AdvancesAAASSearchScience AdvancesMenu

## Supplementary Materials

**This PDF file includes:**

- Section S1. Perturbation theory analysis
- Section S2. Quality factor variation versus surface roughness
- Section S3. Surface morphology of HfO2
- Fig. S1. Measured and simulated WGM resonances in a microtubular cavity.
- Fig. S2. Quality factor variation (*QT*/*Q*0) as a function of surface roughness.
- Fig. S3. Surface morphology of HfO2 characterized by SEM and AFM.
- Reference (*35*)

Download PDF

**Files in this Data Supplement:**

- Adobe PDF - aax6973\_SM.pdf
