# Supplementary material for: Water nanostructure formation on oxide probed in situ by optical resonances
Source: Sci Adv. 2019 Oct 25;5(10):eaax6973. doi: 10.1126/sciadv.aax6973 (PMC6814375; doi:10.1126/sciadv.aax6973)
Supplement: Download PDF [file aax6973_SM.pdf]

Science Advances
AAAS

advances.sciencemag.org/cgi/content/full/5/10/eaax6973/DC1

Supplementary Materials for

Water nanostructure formation on oxide probed in situ by optical resonances

Yin Yin, Jiawei Wang, Xiaoxia Wang, Shilong Li, Matthew R. Jorgensen, Junfeng Ren, Sheng Meng*
Libo Ma*, Oliver G. Schmidt

*Corresponding author. Email: smeng@iphy.ac.cn (S.M.); l.ma@ifw-dresden.de (L.M.)

Published 25 October 2019, *Sci. Adv.* **5**, eaax6973 (2019)
DOI: 10.1126/sciadv.aax6973

This PDF file includes:

Section S1. Perturbation theory analysis
Section S2. Quality factor variation versus surface roughness
Section S3. Surface morphology of HfO_2
Fig. S1. Measured and simulated WGM resonances in a microtubular cavity.
Fig. S2. Quality factor variation (Q_T/Q_0) as a function of surface roughness.
Fig. S3. Surface morphology of HfO_2 characterized by SEM and AFM.
Reference (*35*)

Section S1. Perturbation theory analysis

For a quantitative analysis for the detection of molecular layers, theoretical calculations are performed to simulate the optical resonances in the ring resonator *(18)*. The experimental values of the tube diameter, tube wall thickness, as well as the refractive index of the tube wall are used in a two-dimensional ring resonator model. The calculated mode energies are in good agreement with the experimental data, as shown in fig. S1A. Figure S1B shows the simulated electric field distribution for resonant mode number m = 38.

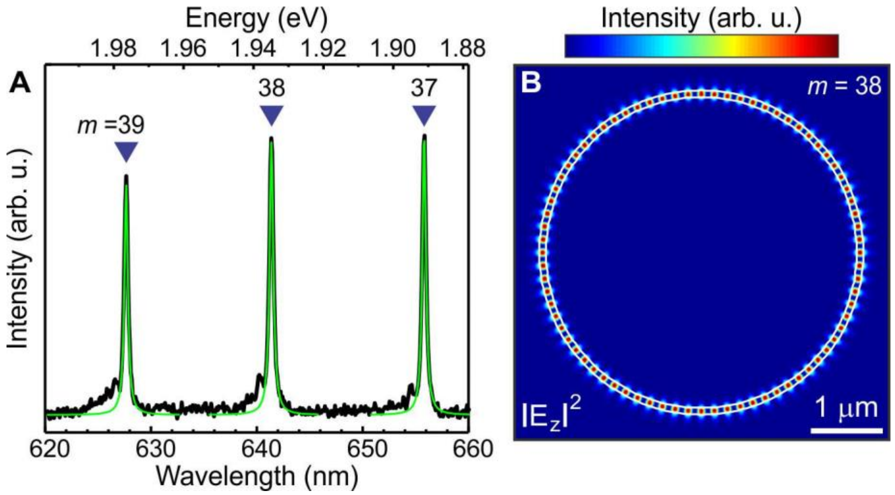

Fig. S1. Measured and simulated WGM resonances in a microtubular cavity. (**A**) Optical resonant modes m = 37-39 are fitted by numerical calculations based on a ring resonator model. The calculation results (blue triangular symbols) are in good agreement with experimental results. (**B**) Calculated electric field profile of mode m = 38 which was selected for sensing measurements.

Based on this simulation data, a perturbation theory analysis is carried out to calculate the shift of mode *m* = 38 induced by the presence of a molecular layer on the tube surface. Thin molecular layers on the inner and outer tube surfaces having the same thickness are considered. Since the layers thicknesses are much smaller than the resonator diameter, perturbation theory is a suitable tool to analyze this system *(20)*. The refractive index of the ice layer is assumed as 1.31. The calculation results are shown in Fig. 2B. Using this method, the thicknesses (h) of adsorbed water layers are calculated. The number of water monolayers is estimated as h/c, where c ~ 0.3 nm is the thickness of a water monolayer.

Section S2. Quality factor variation versus surface roughness

In theory (*35*), the quality factor (Q) of optical resonances is mainly determined by

$$1/Q = 1/Q_s + 1/Q_I \qquad \text{(S1)}$$

where $1/Q_s$ denotes scattering losses caused by surface roughness and $1/Q_I$ (related to optical radiative losses and material losses) is invariant in our experiment. The Q_s is calculated assuming Rayleigh scattering of light due to molecular-sized surface roughness under total internal reflection conditions (*35*)

$$Q_s = \lambda^2 D/2\pi^2\sigma^2 B \qquad \text{(S2)}$$

where σ is the surface roughness, B is the correlation length of the surface roughness, λ is the wavelength, and D is cavity diameter. The correlation length characterizes the spatial “hill”/“valley” variation on the tube surface, which is determined by both the “hill” size and its density distribution on the surface. The initial surface roughness and correlation length can be obtained from the atomic force microscopy (AFM) measurement in Section 3, where the correlation length ~10 nm was used to analytically reveal the Q-factor variation upon roughness. One should note that the correlation length only determines how fast/slow the Q-factor varies upon roughness changes, and does not influence the discussion and conclusion in the main text with numerical calculations. The calculated Q-factor variations are plotted as a function of surface roughness in fig. S2. The curve shows the Q-factor variation trend dependent on surface roughness, where the roughness influenced other losses (e.g. radiative and adsorption) are ignored.

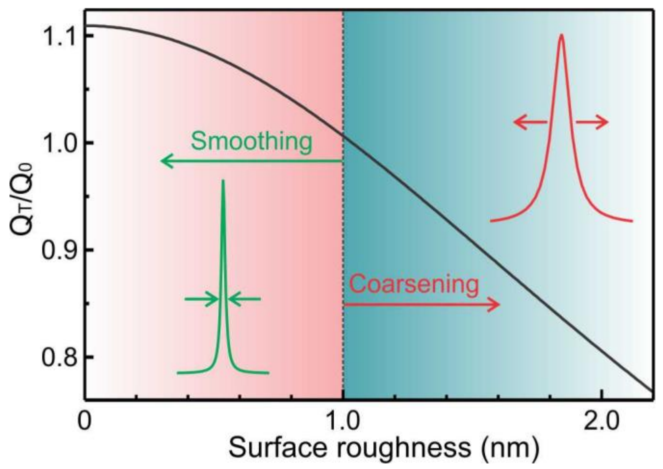

Fig. S2. Quality factor variation (Q_T/Q_0) as a function of surface roughness. The initial surface roughness is ~ 1 nm, where the corresponding quality factor is Q_0. The surface coarsening/smoothing leads to a decrease/increase of the quality factor (QT), which corresponds to the widening/narrowing of the mode width.

Section S3. Surface morphology of HfO_2

Figure S3 shows the experimental characterizations of the tube surface. Both scanning electron microscopy (SEM) and AFM measurements indicate the HfO_2 surface is compact and relatively smooth. The roughness of the HfO_2 surface is characterized as around 1.13 nm (see fig. S3C). In our experiment, a total water film (~5.4 nm) was adsorbed on this surface, and the water film surface is found to be smoother than that of HfO_2.

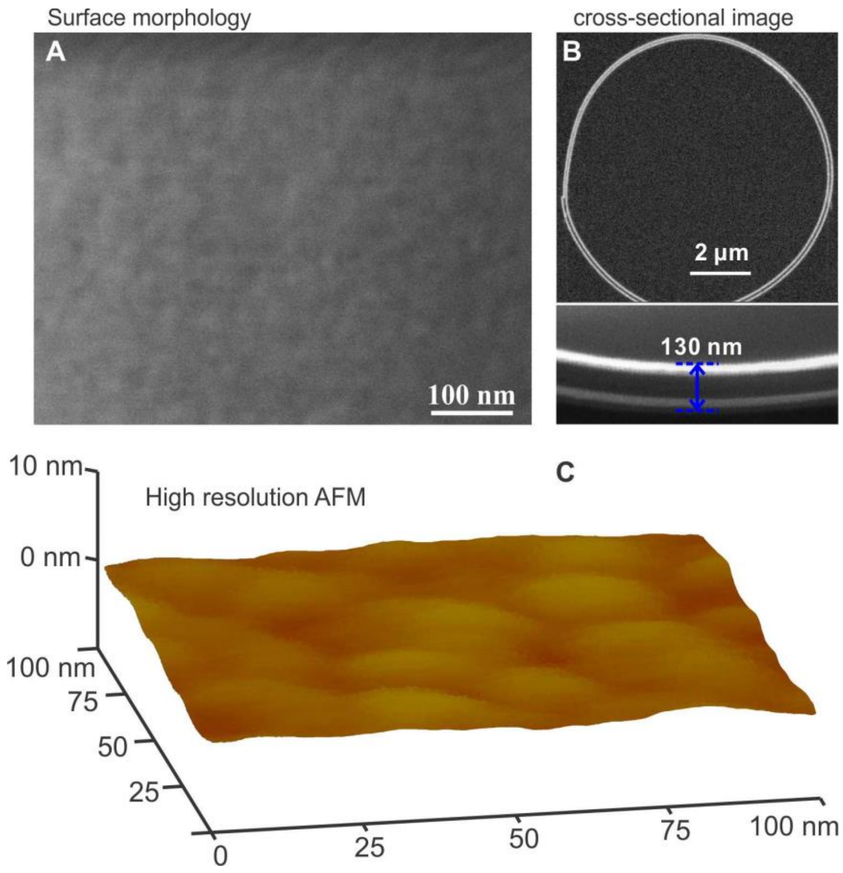

Fig. S3. Surface morphology of HfO_2 characterized by SEM and AFM. (**A**) SEM measurements of our HfO_2 surface and (**B**) the cross-sectional image of our rolled-up tube, where the outer white traces represent HfO_2. (**C**) AFM measurement on HfO_2 surface showing a roughness of 1.13 nm.
